# Emerging Trends in the Management of Gastric Malignancy with Peritoneal Dissemination: Same Disease, Heterogeneous Prognosis

**DOI:** 10.3390/cancers17010117

**Published:** 2025-01-02

**Authors:** Evgenia Mela, Andreas Panagiotis Theodorou, Despina Kimpizi, Kyriaki Konstantinou, Nektarios Belimezakis, Dimitrios Schizas, Dimitrios Theodorou, Tania Triantafyllou

**Affiliations:** 1 First Propaedeutic Department of Surgery, National and Kapodistrian University of Athens, Hippocration General Hospital, 11527 Athens, Greece; antheo300@gmail.com (A.P.T.); despinakimpizi@yahoo.com (D.K.); kyriaki@live.co.uk (K.K.); belimezakisnektarios@gmail.com (N.B.); dimitheod@netscape.net (D.T.); t.triantafyllou89@gmail.com (T.T.); 2 First Department of Surgery, National and Kapodistrian University of Athens, Laikon General Hospital, 11527 Athens, Greece; schizasad@gmail.com

**Keywords:** stage IV, advanced, gastric cancer, peritoneal metastasis, CY1P0, management

## Abstract

Gastric cancer is a significant global contributor to cancer-related mortality. Stage IV gastric cancer represents a significant percentage of patients in Western countries, with peritoneal dissemination being the most prevalent site. Peritoneal disease comprises two distinct entities, macroscopic (P1) and microscopic (P0CY1), which are associated with poor long-term survival rates. Although the present standard of treatment is palliative chemotherapy, a global controversy has arisen concerning specific patients with limited disease burden or conversion to negative lavage cytology following chemotherapy. Available approaches include systemic or intraperitoneal chemotherapy, upfront gastrectomy, and conversion surgery. This review consolidated the current evidence regarding multimodal management, indicating prolonged survival for this distinct subgroup of patients. Considering the complexity of peritoneal metastases, the potential of the multimodal approach unveils promising prospects for identifying the optimal treatment for this particular subset of stage IV patients and thus enhancing their survival outcomes.

## Simple Summary

Gastric cancer is a major health concern globally, contributing substantially to cancer-related mortality. Despite advances in cancer diagnosis and treatment, many patients present with late-stage disease, limiting their treatment options and prognosis. The peritoneum is a frequent site of metastasis. Recent research has focused on the use of a multimodal approach, which combines systemic and/or intraperitoneal chemotherapy with surgery, as a potential alternative to standard palliative chemotherapy. This review focuses on present and emerging advances in the management of gastric cancer patients with peritoneal metastases and indicates that peritoneal dissemination incorporates diverse subgroups of patients with heterogeneous prognoses.

## 1. Introduction

Gastric cancer (GC) is the fifth most prevalent cancer and the fourth-leading cause of cancer-related mortality worldwide [1]. In 2020, approximately 1.1 million cases were diagnosed, resulting in 770,000 deaths globally [1]. GC exhibits significant geographical variability in incidence, with elevated rates observed in Eastern Asia relative to Western nations [1]. Despite a progressive reduction in the incidence of GC, attributable to dietary alterations, tobacco smoking cessation, and Helicobacter pylori eradication, in Western European countries and North America, as well as current improvements in high-risk populations, the global burden on public health remains substantial [2]. Significant differences are also displayed regarding the prognosis of GC among the East and West due to the lack of widespread screening programs in Western countries with lower prevalence of the disease [3]. In contrast, in the East, the introduction of systematic screening programs has led to greater rates of detection at early stages, with documented percentages nearing 50% of new cases [3]. Given the absence of specific symptoms in the early stages, rates of stage IV disease during initial assessment reach 36% [4].

Stage IV GC encompasses distant hematogenous metastases alongside non-regional lymph node metastases and macro- or microscopic peritoneal dissemination. The liver represents the most prevalent site of metastasis, followed by the peritoneum, lung, and bone [5]. Regardless of advancements in medical treatment, the median overall survival for stage IV patients does not exceed 13–16 months [6,7]. However, a distinct subgroup of stage IV GC is emerging under the term of oligometastatic gastric cancer, which includes modest disease burden stage IV GCs [8]. Oligometastatic cancer, according to growing evidence, displays survival benefit (31 months) when treated with an aggressive multimodal strategy combining systemic therapy and local ablative treatment, which surpasses the predicted median survival time of polymetastatic GC undergoing chemotherapy [9]. Nevertheless, chemotherapy serves as the mainstay of treatment according to the European and Japanese Gastric Cancer Guidelines [10,11]. Despite extensive research on systemic treatments, radiotherapy, surgery (gastrectomy or bypass surgery), and palliative approaches, the optimal approach for stage IV GC has yet to be defined [10,11].

Another category requiring further investigation is the lack of macroscopic peritoneal dissemination (P0) accompanied by positive lavage cytology (CY1) as proven during staging laparoscopy, which is classified as stage IV GC according to the UICC, AJCC, and the Japanese system [5,12,13]. Although it has long been established that CY1 is the most substantial indicator of poor survival, increasing evidence suggests that the prognosis of CY1 disease is rather favorable, provided the systemic treatment response and conversion to negative cytological examination results followed by radical gastrectomy [14,15]. Currently, CY1P0 disease represents a global controversy in terms of optimal treatment pathways, necessitating a standardized approach.

The aim of this review is to consolidate the current evidence concerning the diagnosis and treatment of stage IV GC with peritoneal dissemination, emphasizing the current landscape and future prospects.

## 2. Diagnosis and Staging

Subsequent to the diagnosis of GC utilizing endoscopy of the upper gastrointestinal tract and histological examination of biopsies, a multimodal preoperative evaluation for disease staging is warranted [10]. Multidetector computed tomography (MDCT) scans with gaseous and hydrodistension of the stomach serve as the gold standard modality for the preoperative GC staging, with a reported accuracy of 85.7–93% for advanced stages (T3-T4) and 71–78% for overall lymph node (N) staging [16,17]. Nonetheless, endoscopic ultrasound (EUS) exhibits statistically significant greater sensitivity for N staging (91%) in comparison to MDCT (77%), yet both modalities lack specificity (49% and 63%, respectively) [18]. MDCT likewise proves inadequately sensitive for identifying peritoneal disease, thus requiring dependence on secondary signs, including ascites [19,20]. In this context, 18F-FDG PET/CT is not routinely recommended, according to European Society for Medical Oncology (ESMO) guidelines, for GC staging due to low sensitivity for identifying distant metastases and, in particular, peritoneal carcinomatosis (33% and 7%, respectively) [10,21]. In addition, 18F-FDG PET/CT lacks accuracy for mucinous and diffuse tumors due to limited 18F-FDG tracer absorption [10,21].

Given the aforementioned limitations of imaging methods for diagnosing peritoneal metastases, staging laparoscopy (SL) has been established in the preoperative examination of GC patients for occult peritoneal dissemination [20]. In Western nations, where rates of stage IV GC exceed 30% at initial diagnosis, the ESMO guidelines recommend performing SL in all patients with stage IB or higher who are potentially eligible for perioperative chemotherapy [4,10]. Conversely, in the East, in accordance with the 6th edition of the Japanese Gastric Cancer Treatment Guidelines, there is a weak indication for patients diagnosed in advanced stage and who display high-risk indicators, including linitis plastic, Bormann type 3–4, and poorly cohesive tumors [11]. Ramos et al.’s metanalysis demonstrated an overall sensitivity of 84.6% and specificity of 100% for SL [22]. Likewise, Leake et al. reported comparable outcomes with an overall accuracy for M staging of 85–98.9%, whereas SL altered the treatment plan in 8.5–59.6% of patients, resulting in 8.5–43.8% less laparotomies [23].

The Peritoneal Carcinomatosis Index (PCI) is a numerical score that macroscopically evaluates the extent of peritoneal disease during SL. The abdominal cavity is separated into 13 sections, and the size and distribution of peritoneal lesions are examined to calculate a score ranging from 0 to 39 [20,24]. The optimal cut-off for surgical PCI score for determining oligometastatic disease, which may benefit from aggressive local treatment, remains ambiguous. Notably, low PCI scores enable complete cytoreduction, which is the primary determinant of survival rates. Although Glehen et al. recommended a cut-off of 12, subsequent studies suggest that PCI < 7 presents a favorable survival [25,26,27]. An additional score for estimating the degree of peritoneal metastasis is the classification into three grades (P1a, P1b, P1c) according to the 15th Japanese classification system, with the limitation of the inability to evaluate the magnitude and quantify the dissemination of the peritoneal lesions [13,24]. The implementation of lavage cytology, while performing SL, enables the detection of occult peritoneal carcinomatosis, which represents a recurrence predictor with reported recurrence rates of 11.1–100% for positive (CY1) and 0–51% for negative patients (CY0) [23].

In addition, magnetic resonance imaging (MRI) exhibits an emerging role in GC staging. Particularly for peritoneal disease, in the systematic review of Laghi et al. MRI was compared to CT in terms of sensitivity (88% vs. 86%) and specificity (86% vs. 83%) in GC patients, albeit the difference was not statistically significant [28]. Nevertheless, in 2023, the subsequent systematic review of Ho et al. showed that MRI displays greater sensitivity and accuracy by an average rate of 38.1% and 33.75%, respectively, whereas CT has equivalent or higher specificity [29].

Considering the complex nature of GC, artificial intelligence is also arising as a supplementary tool, with potential applications in predicting peritoneal dissemination. In 2021, Jiang et al. devised a deep-learning model, the Peritoneal Metastasis Network, using CT scans to predict occult peritoneal disease. The proposed model yielded 87.5% sensitivity, 92.9% specificity, and an area under the curve (AUC) of 0.92, thus surpassing clinical variables with an AUC of 0.51–0.63 [30]. Similarly, Chinese experts developed a multimodal machine-learning model, the MMF model, to estimate peritoneal metastasis and demonstrated that the combination of PET/CT features with clinical variables and experts’ opinions delivers greater results than each variable alone. The MMF model exhibited a good discriminative performance with an AUC of 0.908, which was maintained at 0.897 in the subgroup of mucinous and signet ring cell tumors [31]. However, regardless of the promise of MRI and AI applications in GC staging, further multicenter studies are required to enable their introduction into regular clinical practice.

Recent advances in molecular profiling of GC have not only offered insight into the carcinogenesis pathway but also provided novel methods for diagnostic and therapeutic purposes [10,11]. In particular, current validated and established biomarkers for advanced and metastatic GC include human epidermal growth factor receptor 2 (HER2) expression via immunochemistry or in situ hybridization, programmed cell death ligand 1 (PD-L1) expression via immunochemistry and microsatellite instability [10]. However, the rates of HER2 positivity vary by metastatic site, with a significantly higher prevalence in GC patients with liver metastasis (80%) compared to peritoneal dissemination (16.4%) [32]. In addition, it has long been established that intestinal and diffuse type GC are inherently distinct entities [33]. The diffuse-type carcinomas, which metastasize more frequently to the peritoneum, demonstrate genomic stability, while microsatellite instability related to intestinal-type histology is associated with improved response to immunotherapy [34]. Thus, the histology, according to the Lauren classification and the molecular biology of the tumor, should always be examined in advanced and metastatic settings in order to tailor the treatment correspondingly [10].

## 3. Treatment

### 3.1. Systemic Therapy

Multidisciplinary discussion for treatment planning prior to any treatment decision is indispensable for all patients. Palliative systemic therapy constitutes the standard treatment for metastatic GC according to the NCCN and ESMO guidelines, as it significantly ameliorates the survival and the quality of life when compared to optimal supportive care. A first-line chemotherapy regimen includes a platinum/fluoropyrimidine doublet or triplet when a third agent, such as docetaxel, is added [10]. In terms of fluoropyrimidines, oral capecitabine is an equivalent, well-tolerated alternative to infusional 5-fluorouracil featuring different toxicity profiles [10,35]. Molecular profiling has facilitated the incorporation of novel targeted therapies in the management of metastatic GC. HER2 is an established treatment target for advanced and metastatic GC [10,11]. In 2010, the phase III ToGA trial proved the statistically significant benefit of combining trastuzumab with cisplatin plus 5-fluorouracil (5-FU) in terms of overall survival (OS) and progression-free survival (PFS) for HER2-positive metastatic gastric or gastroesophageal cancer patients [36]. A recent phase II randomized trial assessed trastuzumab deruxtecan, which is an antiHER2 antibody–drug conjugate with a cytotoxic topoisomerase I inhibitor, and reported significant efficacy for HER2-positive patients who progressed on trastuzumab [37]. However, the outcomes of subsequent trials evaluating other second-line antiHER2 antibodies, including lapatinib and trastuzumab emtansine [38,39], were unfavorable. Concurrently, depending on the findings of the REGARDS and RAINBOW trial, the administration of an anti-vascular endothelial growth factor receptor 2 (VEGFR2), ramuricumab, combined with paclitaxel has also been incorporated in the second-line treatment recommendations for metastatic GC [10,40,41].

The advent of immune checkpoint inhibitors altered the therapeutic management of metastatic GC. The phase III CheckMate 649 trial introduced the addition of immunotherapy, using nivolumab, to chemotherapy for metastatic HER2 negative GC patients with a PD-L1 expression of CPS ≥ 5 [42]. Pembrolizumab is another immune checkpoint inhibitor administered as a second-line treatment in microsatellite instability-high (MSI-H) metastatic settings [10]. Despite the promising outcomes that it displayed in the phase II KEYNOTE-059 trial, in the subsequent phase III KEYNOTE-062 trial, pembrolizumab monotherapy or plus chemotherapy did not outperform chemotherapy in terms of OS and PFS [43,44]. However, in KEYNOTE-061, it displayed benefits as a second-line treatment for MSI-H advanced GC patients, which was further confirmed in the KEYNOTE-158 trial [45,46]. In light of the established understanding that trastuzumab enhances PD1 and PD-L1 tumor expression, the phase III KEYNOTE-811 trial recruited patients with metastatic HER2-positive gastric or gastroesophageal junction cancer and compared pembrolizumab to placebo always coupled with trastuzumab and chemotherapy. Recently, the final analysis was presented at ESMO Congress 2024, highlighting a significantly superior objective response rate for pembrolizumab relative to placebo (72.6% vs. 60.1%) and significantly prolonged OS and PFS, with greater benefit in OS for patients with CPS ≥ 1 [47]. While novel systemic treatments, including targeted therapy and immunotherapy, have emerged for the management of metastatic GC, there is still a lack of evidence regarding survival improvement for P1 and P0CY1 GC patients. Additional multicenter prospective studies and controlled trials are needed to determine the optimal systemic treatment for this distinct subgroup of stage IV GC.

### 3.2. Surgery

Currently, radical gastrectomy with D2 lymphadenectomy represents the standard of care for resectable GC, while D1 or D1+ lymphadenectomy is recommended for early gastric cancer (T1) with no nodal metastasis [10,11]. Gastrectomy with D2 extended lymphadenectomy is considered, according to the Japanese guidelines, as a non-standard resection for GC patients with bulky regional nodal disease following neoadjuvant therapy [11]. However, despite its feasibility and safety, further large-scale prospective studies are needed to establish its benefit for advanced gastric cancer [11,48]. In addition, extensive intraperitoneal lavage has been advocated as a preventive strategy for peritoneal metastases in advanced GC patients undergoing curative gastrectomy. Yet, a recent meta-analysis did not yield a significant advantage for overall survival and peritoneal recurrence-free survival [49]. Furthermore, cytoreductive surgery, which has been examined and performed at experienced centers, aims to eradicate all macroscopic primary and peritoneal diseases by gastrectomy, D2 lymphadenectomy, and peritonectomy according to the Sugarbaker methods [50]. As previously stated, both P1 and P0CY1 are considered stage IV. As a consequence, surgical resection is not offered to patients with macroscopic or microscopic peritoneal disease under standard treatment pathways and is reserved for highly selective patients in Western countries, considering its controversial biological basis in metastatic settings [10].

Concerning P0CY1 patients, the retrospective study of Mezhir et al. showed no significant difference in disease-specific survival among patients who received either chemotherapy (1.7 years) or gastrectomy (1.1 years) [51]. Likewise, the multicenter randomized controlled trial “REGATTA” (JCOG0705/KGCA01) enrolled 175 patients with a single non-curable variable and did not demonstrate improved survival from reduction surgery over chemotherapy alone [52]. Conversely, in the preceding single-arm (CCOG0301) trial in Japan, 47 CY1 GC patients underwent radical gastrectomy followed by S-1 adjuvant chemotherapy and achieved a median survival time of 705 days and 5-year OS of 26% [53]. In several retrospective studies, P0CY1 patients demonstrate an improved prognosis compared to P1 GC patients [54,55].

In this context, numerous studies have documented the survival advantage of individuals converting from positive to negative cytology subsequent to upfront systemic chemotherapy followed by surgery. The introduction of conversion surgery for GC patients was initially delivered by Nakajima et al. in 1997 and is identified as surgery with the objective of R0 resection following systemic treatment for tumors initially regarded as inoperable or borderline inoperable [56]. Aizawa et al. displayed statistically significant improvement in 5-year OS for the converted group (34.6%) in comparison to the non-converted group (17.6%) [57]. Yasufuku et al., Yago et al., and Yamaguchi et al. reported comparable findings, revealing a statistically significant benefit in 3-year OS (76.9% vs. 10.7%) and (75% vs. 16.7%), as well as median survival time (32 months vs. 18.8 months) for patients with converted lavage cytology, respectively [15,58,59]. Furthermore, in the prospective study conducted by Li et al., patients who had conversion surgery exhibited greater OS relative to those who underwent upfront surgery. Nonetheless, there is currently a lack of prospective evidence regarding the optimal systemic treatment regimen for peritoneal dissemination in GC [60]. In Nakayama et al.’s retrospective study, P0CY1 GC patients were treated with S-1 monotherapy or combined therapy of S-1 with cisplatin and reported no significant difference in median OS and PFS. However, S1 monotherapy was administered prior to the release of the SPRITS study results [61]. Valletti et al., in a subsequent retrospective study, reported a significantly greater conversion rate with the FLOT regimen (5-FU, leucovorin, oxaliplatin, and docetaxel) in comparison to ECF (epirubicin, cisplatin, and 5-FU), with the limitation, though, of a small sample of CY1 patients who converted to CY0 [62]. An additional interesting finding is the recurrence pattern, with peritoneal recurrence representing the most prevalent in CY1 patients, with documented rates varying between 55 and 78% [51,53,58,62].

Regarding P1 patients, multiple retrospective research studies on a rather small sample of patients have assessed the impact of multimodal therapy integrating surgery and systemic chemotherapy, the majority of which indicate that the effectiveness of surgical intervention following chemotherapy is significantly superior to chemotherapy alone. Specifically, Okabe et al. observed a statistically significant difference in median survival time between patients who underwent curative resection (43.2 months) and chemotherapy alone (10.3 months) [63]. In a similar way, two subsequent studies displayed a significant survival benefit for patients treated with conversion surgery [64,65]. In contrast, Valletti et al. presented a 16-month median survival time for P1 patients who were treated with a combination of chemotherapy and surgical resection [62]. Table 1 and Table 2 consolidate the results for P0CY1 and P1 patients, respectively.

### 3.3. Intraperitoneal Therapy

Despite the predominant role of systemic chemotherapy in treating stage IV GC, its efficiency is hampered by the peritoneal plasma barrier [66]. The introduction, thus, of intraperitoneal chemotherapy addresses the need for elevated regional levels of chemotherapeutic agents while aiming to minimize systemic cytotoxic adverse effects. The phase II CY-PHOENIX trial enrolled 38 CY1 patients who received combined intraperitoneal and intravenous chemotherapy, which resulted in 84.2% 1-year OS [67]. Although the phase III PHOENIX-GC study did not yield a statistical survival benefit for intraperitoneal paclitaxel coupled with systemic chemotherapy, the patients had an increased burden of peritoneal metastases, with only six patients out of the one hundred sixty-four patients being P1 according to the Japanese Classification of Gastric Carcinoma [68]. Saito et al., in twenty patients who underwent debulking surgery following intraperitoneal and intravenous chemotherapy, indicated a 100% 1-year OS in contrast to the 62.5% of the non-surgery subgroup (*p* < 0.01) [69].

Hyperthermic intraperitoneal chemotherapy (HIPEC) constitutes an alternative approach exploiting the advantages of regional cytotoxicity of both chemotherapy and hyperthermia. In 2011, a phase III randomized controlled trial studied the difference between cytoreductive surgery alone versus cytoreductive surgery with HIPEC in the Asian population. Both subgroups had a median PCI of 15, and combination therapy surpassed cytoreductive surgery alone with a median survival time of 11 months versus 6.5 months [70]. The subsequent phase III GYMSSA was conducted on the Western population, which differs from the Eastern, and was terminated at early stages due to difficulty acquiring patients and increased complication (44%) and mortality rates (11%). Out of the 17 patients who participated in the study, the cytoreduction subgroup experienced 11.3 months median OS, while the standard chemotherapy subgroup exhibited 4.3 months median OS [71]. Badgwell et al. conducted a phase II randomized controlled trial administering systemic chemotherapy in addition to laparoscopic HIPEC, and the patients with treatment response underwent surgical resection. The morbidity and mortality rates were reduced in comparison to GYMSSA’s, and the referred 3-year OS rate from the initial stage IV disease was 43.5% [72]. Researchers from the same institution, in a following phase II trial, enrolled patients who had received intravenous chemotherapy paired with at least one cycle of laparoscopic HIPEC and implemented cytoreductive resection, gastrectomy, and HIPEC and reported a 70% morbidity rate, 0% mortality rate, and a 3-year OS of 28% [73]. Similarly, in a prospective European cohort study, patients with PCI < 6 achieved promising survival outcomes with a median OS of 19 months [74]. Concurrently, the multicenter French retrospective CYTO-CHIP study observed a 6-month survival benefit in the HIPEC group and a 5-year recurrence-free survival rate of 20%, while it concluded that in individuals receiving HIPEC PCI values < 7 and <13 for non-poorly cohesive and for poorly cohesive carcinomas, respectively, had predictive value for OS [75]. Recently, the phase III GASTRIPEC-I study, which compared perioperative chemotherapy and cytoreductive surgery to HIPEC and cytoreductive surgery, demonstrated no significant OS deviation, although the HIPEC arm exhibited superior metastasis-free and progression-free survival. This lack of statistical significance for OS was attributed to the extent of peritoneal disease, with 44% of patients displaying PCI > 7 and 40% ascites [76].

Pressurized intraperitoneal aerosol chemotherapy (PIPAC) is implemented laparoscopically and represents an alternative emerging approach for intraperitoneal chemotherapy administration, asserting repeatability and enhanced drug distribution and penetration. The utilization of PIPAC as a drug delivery method has primarily been investigated in Europe for the treatment of patients with a high burden of peritoneal metastases with palliative intent. In 2018, Struller et al., in a single-arm phase II study, enrolled 25 P1 patients and demonstrated a median OS of 6.7 months following treatment with three cycles of PIPAC with cisplatin and doxorubicin and 6-week intervals [77]. In a subsequent retrospective study, 42 patients with a median PCI of 17 received PIPAC with cisplatin and doxorubicin coupled with systemic chemotherapy and had a remarkable median OS of 19.1 months [78]. A recent phase I trial combined oxaliplatin PIPAC with intravenous nivolumab for 19 patients with a median PCI of 20 and reported a PCI decrease by 5 and 7 following the second and third PIPAC, respectively. Concurrent translational analysis revealed a modified tumor microenvironment with enhanced T-cell infiltration resulting from the combination of immunotherapy and regional therapy. Despite the lack of comparative studies, PIPAC exhibits encouraging results for high-volume peritoneal dissemination with an acceptable safety profile [79]. Table 3 summarizes the findings of the aforementioned studies.

## 4. Future Perspectives

The present landscape suggests that a multimodal approach combining systemic and regional therapy for selected GC patients with peritoneal disease yields encouraging outcomes. Ongoing clinical trials aim to address the arising challenges and define the group of patients who display the greater benefit. Regarding oligometastatic gastric cancer aside from peritoneal, curative intent surgery subsequent to response to systemic chemotherapy is examined in the French SURGIGAST and the German AIO-FLOT5 (“RENAISSANCE”) trials, with the primary endpoint being the OS. Specifically, the two phase III randomized controlled trials aim to determine if surgery paired with palliative chemotherapy or perioperative FLOT treatment, respectively, improves survival rates over palliative chemotherapy alone [80,81]. The final analysis of the RENAISSANCE trial was recently presented at ASCO Congress 2024, revealing that patients with peritoneal disease displayed a negative effect on the median OS of 12 months in the surgery arm in contrast to 19 months in the palliative chemotherapy arm, thus advocating against surgical resection for peritoneal disease [82]. Concerning intraperitoneal treatments, the phase III PERISCOPE II trial investigates whether gastrectomy combined with cytoreductive surgery and HIPEC will outperform palliative systemic treatment in terms of OS in GC patients with macroscopic (PCI < 7) and/or microscopic peritoneal disease [83]. Concurrently, the ongoing phase II STOPGAP trial is enrolling P1 or P0CY1 GC or Siewert 3 gastroesophageal junction patients to evaluate the safety and the efficacy of repeated systemic and intraperitoneal chemotherapy with paclitaxel [84]. Furthermore, the double-arm GASTRICHIP trial, which launched in 2014, examines the impact of low-dose prophylactic HIPEC with oxaliplatin in GC patients exhibiting at least one of the following variables: T3 or T4, lymph node metastasis, and P0CY1 [85]. For PIPAC, the phase II PIPAC Estok 01 trial attempts to evaluate the PFS and secondary the 2-year OS in P1 patients with PCI > 8 undergoing either PIPAC alternating with systemic chemotherapy or systemic chemotherapy alone [86]. In this context, a single-arm feasibility trial is enrolling P1 GC patients to assess the combination of PIPAC with cisplatin and doxorubicin and systemic chemotherapy [87]. Nonetheless, despite the abundance of potential treatment approaches, there is still a lack of large prospective cohort studies followed by randomized controlled trials in order to compare each strategy, exclusively for P0CY1 and P1 individuals; identify the relevant prognostic indicators for peritoneal treatment in GC; guide patients with heterogeneous prognosis; and determine the optimal treatment tailored to each molecular profile.

## 5. Conclusions

The incidence of peritoneal metastases remains significant during the initial diagnosis of GC and is linked to poor prognostic outcomes. CT scans constitute the standard of care in GC diagnosis, while SL is recommended in stages IB or higher. Modern chemotherapeutic agents and targeted therapies have resulted in the extension of some of these patients’ lifespans, allowing for further investigations and comparisons between treatment options. Although a rising number of studies have highlighted that multimodal therapy comprising systemic and intraperitoneal chemotherapy along with surgical resection yields favorable overall survival rates in selective patients, the current standard treatment remains palliative chemotherapy. These findings indicate that the different biologic characteristics of patients with limited disease burden, adequate treatment response, or expression of immunotherapy receptors suggest that they are a distinct subpopulation that is not currently subdivided by the existing stage IV TNM classification. Therefore, further clinical trials are required to examine the veiled optimism on the significance of clinical equipoise and determine more accurately the subgroup of patients who display greater survival benefit from multimodality treatments.

## Figures and Tables

**Table 1:** CY1 GC patients.

Author	Publication Year	Country	Type of Study	Number of Patients	Treatment	Outcomes
Kodera et al. [53]	2009	Japan	Phase II	47	Gastrectomy followed by S1	MST 705 days, PFS 376 days 2-year survival rate: 46% 5-year survival rate: 26% Peritoneal recurrence: 62%
Mezhir et al. [51]	2010	US	Retrospective	93	Gastrectomy (29 patients) vs. chemotherapy (63 patients)	MST 1.1 year for gastrectomy group and 1.7 years for chemotherapy group Peritoneal recurrence 69% Median DSS 2.5 years for converted group (*n* = 27) vs. 1.4 years for non-converted group (*n* = 21) (*p* = 0.0003)
Lee et al. [54]	2012	Korea	Retrospective	172	Chemotherapy ± gastrectomy	MST 20 months for P0CY1 and 10 months for P1CY1
Badgwell et al. [55]	2008	US	Retrospective	39	Neoadjuvant treatment ± gastrectomy	MST 12.8 months for P0CY1 and 10.2 months for P1
Aizawa et al. [57]	2015	Japan	Retrospective	47	SP, DCS, FCP ± conversion surgery	MST 20.4 months 5-year survival rate: 25% Converted group: MST 30.4 months, 5-year survival rate 34.6% Non-converted group: MST 15 months, 5-year survival rate 17% (*p* = 0.03)
Fujitani et al. [52]	2016	Japan and Korea	Phase III	175	SP vs. gastrectomy + adjuvant SP	MST 16.6 months for chemotherapy-alone group vs. 14.3 months for gastrectomy plus chemotherapy group (*p* = 0.70)
Nakayama et al. [61]	2017	Japan	Retrospective	44	S1 (*n* = 25) or SP (*n* = 19) + gastrectomy	S1 group: MST 28.2 months, PFS 15.6 months SP group: MST 24.0 months, PFS 18.8 months
Yasufuku et al. [15]	2020	Japan	Retrospective	39	Chemotherapy ± gastrectomy vs. gastrectomy	MST: 108.5 months for the converted group 16.3 months for the non-converted group undergoing palliative chemotherapy 18.0 months for the initial surgery group (*p* = 0.0005) 3-year survival rate: 76.9% for the converted group 10.7% for the non-converted group undergoing palliative chemotherapy 0% for the surgery initial group
Yamaguchi et al. [59]	2021	Japan	Multicenter retrospective	713	Chemotherapy (SP or DCS or other) ± gastrectomy vs. gastrectomy	MST 32 months for the converted group vs. 18.8 for the non-converted group (*p* = 0.001) MST 32 months for the converted group vs. 24 months for the initial surgery group (*p* = 0.004)
Valletti et al. [62]	2021	Switzerland	Retrospective	172	FLOT or ECF + gastrectomy	Negative for PM (*n* = 125): 3-year survival rate 65% Peritoneal recurrence 25% Converted group (*n* = 9): 3-year survival rate 53% Peritoneal recurrence 55% Non-converted CY1 group (*n* = 5): MST 13 months 1-year DFS 20%
Yago et al. [58]	2022	Japan	Retrospective	38	Chemotherapy ± gastrectomy vs. gastrectomy	MST 19.7 months for initial chemotherapy group vs. 17.7 months for initial surgery group (*p* = 0.569) Peritoneal recurrence 78.1% 3-year survival rate: 75% for the converted group 16.7% for the non-converted group undergoing palliative chemotherapy 0% for the non-converted group undergoing palliative gastrectomy
Li et al. [60]	2023	China	Prospective	96	Chemotherapy ± gastrectomy vs. gastrectomy	MST 36.1 months for initial chemotherapy group vs. 29.7 for initial surgery group (*p* = 0.367) PFS 18.1 months for initial chemotherapy group vs. 16.1 in initial surgery group (*p* = 0.861)

MST: median survival time; PFS: progression-free survival; DSS: disease-specific survival; PM: peritoneal metastases; SP: S1 plus cisplatin; 5FU: 5-fluorouracil; DCS: docetaxel plus cisplatin plus S1; FCP: 5FU plus cisplatin plus paclitaxel; PTX: paclitaxel; FLOT: 5FU plus leucovorin plus oxaliplatin plus docetaxel; ECF: epirubicin plus cisplatin plus 5FU.

**Table 2:** P1 GC patients.

Author	Publication Year	Country	Type of Study	Number of Patients	Treatment	Outcomes
Okabe et al. [63]	2009	Japan	Retrospective	41	SP or DS ± surgery	MST 43.2 months for curative resection, 12.6 months for non-curative resection, 10.3 months for chemotherapy alone (*p* < 0.0001)
Yamamoto et al. [64]	2012	Japan	Retrospective	13	SP or DS ± surgery	MST 794 days for surgery, 505 days for chemotherapy alone (*p* < 0.05)
Kim et al. [65]	2014	Korea	Retrospective	43	FP or SP ± surgery	MST 37 months for curative resection, 18 months for non-curative resection, 8 months for chemotherapy alone (*p* < 0.001)
Valletti et al. [62]	2021	Switzerland	Retrospective	172	FLOT or ECF + gastrectomy	Negative group for PM (*n* = 125): 3-year survival rate 65% PM group (*n* = 33): MST 16 months

MST: median survival time; SP: S-1 plus cisplatin; DS: docetaxel plus S-1; FP: 5-fluorouracil plus cisplatin; FLOT: 5-fluorouracil plus leucovorin plus oxaliplatin plus docetaxel; ECF: epirubicin plus cisplatin plus 5-fluorouracil.

**Table 3:** Studies for intraperitoneal therapies.

Author	Name of Study	Publication Year	Country	Type of Study	Number of Patients	Treatment	Outcomes
Yang et al. [70]		2011	China	Phase III	68 P1	Cytoreductive surgery ± HIPEC (cisplatin plus mitomycin C)	MST 11 vs. 6.5 months (*p* = 0.046)
Rudloff et al. [71]	GYMSSA	2014	USA	Phase III	17 P1	Cytoreductive surgery + HIPEC + FOLFOXIRI vs. FOLFOXIRI	MST 11.3 vs. 4.3 months
Aizawa et al. [67]	CY-PHOENIX	2017	Japan	Phase II	38 CY1	Intraperitoneal + intravenous PTX	1-year OS 84.2%
Badgwell et al. [72]		2017	USA	Phase II	19 P1 or P0CY1	Systemic chemotherapy (Taxol-5-FU—oxaliplatin) + laparoscopic HIPEC mitomycin C plus cisplatin ± resection	MST 30.2 months 1-year OS 94.7% 3-year OS 43.5%
Ishigami et al. [68]	PHOENIX-GC	2018	Japan	Phase III	164 P1	Intraperitoneal + intravenous PTX plus S-1 vs. intravenous SP	MST 17.7 vs. 15.2 months (*p* = 0.08) 3-year OS 21.9% vs. 6%
Rihuete Caro et al. [74]		2018	Spain	Prospective	35 P1	Cytoreductive surgery + HIPEC + perioperative systemic chemotherapy	MST 16 months 1-year OS 70.8% 3-year OS 21.3% 5-year OS 21.3%
Struller et al. [77]		2019	Germany	Phase II	25 P1	PIPAC C/D (3 cycles)	MST 6.7 months PFS 2.7 months
Alyami et al. [78]		2021	France	Retrospective	42 P1	PIPAC C/D + systemic chemotherapy	MST 19.1 months
Badgwell et al. [73]		2021	USA	Phase II	20 P1 or P0CY1	Systemic chemotherapy plus laparoscopic preoperative HIPEC + cytoreductive surgery, gastrectomy, HIPEC	MST 22.1 months 1-year OS 90% 3-year OS 28% Peritoneal recurrence 50%
Saito et al. [69]		2021	Japan	Prospective	44 P1 or P0CY1	Intraperitoneal PTX + intravenous SOX ± gastrectomy	MST 25.8 months 1-year OS 79.5% Median PFS 18.2 months
Bonnot et al. [75]	CYTO-CHIP	2021	France	Multicenter retrospective	277 P1	Cytoreductive surgery ± HIPEC	MST 16.7 vs. 11.3 months (*p* = 0.024) for poorly cohesive carcinomas MST 34.5 vs. 14.3 months for non-poorly cohesive carcinomas (*p* = 0.008)
Rau et al. [76]	GASTRIPEC-I	2024	Germany	Phase III	105 P1	Perioperative systemic chemotherapy EOX + cytoreductive surgery vs. cytoreductive surgery + HIPEC (mitomycin plus cisplatin)	MST 14.9 vs. 14.9 months (*p* = 0.165) PFS 3.7 vs. 7.1 months (*p* = 0.047) MFS 9.2 vs. 10.2 months (*p* = 0.028)
Sundar et al. [79]	PIANO	2024	Singapore	Phase I	18 P1	PIPAC-OX and i.v nivolumab	Median PCI was decreased by 5 and 7 at second and third PIPAC-OX. PFS 1.8 months MST 6 months

MST: median survival time; OS: overall survival; MFS: other distant metastasis-free survival; PTX: paclitaxel; SP: S-1 plus cisplatin; SOX: S-1 plus oxaliplatin; EOX: epirubicin oxaliplatin plus capecitabine; FOLFOXIRI: oxaliplatin and irinotecan plus 5-FU; PIPAC C/D: PIPAC with cisplatin and doxorubicin; PIPAC-OX: PIPAC with oxaliplatin.
